# Pre-Existing Hypertension Dominates γδT Cell Reduction in Human Ischemic Stroke

**DOI:** 10.1371/journal.pone.0097755

**Published:** 2014-05-19

**Authors:** Mateusz G. Adamski, Yan Li, Erin Wagner, Hua Yu, Chloe Seales-Bailey, Helen Durkin, Qing Hao, Steven A. Soper, Michael Murphy, Alison E. Baird

**Affiliations:** 1 Department of Neurology, SUNY Downstate Medical Center, Brooklyn, New York, United States of America; 2 Department of Neurology, Jagiellonian University Medical College, Krakow, Poland; 3 Department of Pathology, SUNY Downstate Medical Center, Brooklyn, New York, United States of America; 4 University of North Carolina, Chapel Hill, North Carolina, United States of America; 5 Louisiana State University, Baton Rouge, Louisiana, United States of America; Uniform Services University of the Health Sciences, United States of America

## Abstract

T lymphocytes may play an important role in the evolution of ischemic stroke. Depletion of γδT cells has been found to abrogate ischemia reperfusion injury in murine stroke. However, the role of γδT cells in human ischemic stroke is unknown. We aimed to determine γδT cell counts and γδT cell interleukin 17A (IL-17A) production in the clinical setting of ischemic stroke. We also aimed to determine the associations of γδT cell counts with ischemic lesion volume, measures of clinical severity and with major stroke risk factors. Peripheral blood samples from 43 acute ischemic stroke patients and 26 control subjects matched on race and gender were used for flow cytometry and complete blood count analyses. Subsequently, cytokine levels and gene expression were measured in γδT cells. The number of circulating γδT cells was decreased by almost 50% (p = 0.005) in the stroke patients. γδT cell counts did not correlate with lesion volume on magnetic resonance diffusion-weighted imaging or with clinical severity in the stroke patients, but γδT cells showed elevated levels of IL-17A (p = 0.048). Decreased γδT cell counts were also associated with older age (p = 0.004), pre-existing hypertension (p = 0.0005) and prevalent coronary artery disease (p = 0.03), with pre-existing hypertension being the most significant predictor of γδT cell counts in a multivariable analysis. γδT cells in human ischemic stroke are reduced in number and show elevated levels of IL-17A. A major reduction in γδT lymphocytes also occurs in hypertension and may contribute to the development of hypertension-mediated stroke and vascular disease.

## Introduction

Stroke is a leading cause of death and disability in the United States [1]. The immune system is recognized as playing a major role in the evolution and pathophysiology of stroke and microvascular dysfunction, with a growing number of reports on the role of T lymphocytes [2]–[7].

γδT cells are a population of T lymphocytes that comprise between 0 and 7% of circulating CD3^+^ cells [8]. These cells express γδ T cell receptors (TCR), whereas the majority of T lymphocytes express αβTCR. γδT cells secrete interleukin-17 (IL-17) when stimulated by interleukin-23 (IL-23) and interferon gamma (INF-γ) when stimulated by interleukin-12 (IL-12) [9], [10]. As a bridge cell population γδT cells break the classical immune system paradigm by having features of both the adaptive (e.g., the antigen experienced response) and innate (i.e., the rapid response) immune responses. γδT cells that secrete IL-17 have been characterized as ligand-naive and γδT cells that secrete INF-γ have been characterized as ligand-experienced [9].

In a prior study depletion of γδT cells in mice ameliorated ischemia-reperfusion injury in the brain [11]. When analyzing the mechanistic basis of γδT cells in this model, Shichita et al. [11] identified important roles and interactions of IL-23 and IL-17 but not INF-γ. After ischemia reperfusion injury, increased macrophage IL-23 secretion commenced after day 1 and increased γδT cell IL-17 secretion commenced after day 3. In this same model, infarct size was reduced on day 1 in IL-23 knockout (KO) mice and on day 4 in IL-17 KO mice but was not altered in INF-γ KO mice. Intracellular cytokine staining confirmed that γδT cells were the main source of IL-17, CD4^+^ and γδTCR- T cells were the main sources of INF-γ.

To date there is little data on the role of γδT cells in human stroke [12]. The mice in Shichita et al's study [11] were young and apparently free of vascular risk factors, while older age, a major risk factor for stroke, has been associated with reduced γδT cell counts [13], [14]. Furthermore, hypertension and coronary artery disease, other major stroke risk factors, have been associated with alterations in IL-17 and IFN-γ [15]–[18]. Therefore, our objectives were to determine γδT cell counts and function in the clinical setting of ischemic stroke and the associations of γδT cell counts with lesion volume, measures of clinical severity and with major stroke risk factors. We have investigated whether circulating γδT cell counts are 1) altered in patients presenting with acute ischemic human stroke, and 2) correlate with: lesion volume on diffusion-weighted magnetic resonance imaging (DW MRI), and with clinical severity and demographic and risk factors. Next we examined γδT cell levels of IL-17A and INF-γ and the cellular gene expression of interleukin-23 receptor (*IL-23R*) and interleukin-23 subunit alpha (*IL-23A*).

## Methods

Approval was obtained from the SUNY Downstate Medical Center Institutional Review Board for this prospective observational study. All patients and/or authorized representatives gave full and signed informed consent.

### Study Subjects

The peripheral blood samples of 69 subjects were used: 43 acute stroke patients admitted to the University Hospital of Brooklyn at SUNY Downstate Medical Center and at Long Island College Hospital, and 26 gender and race matched healthy control subjects recruited from the local community. The clinical and laboratory characteristics of patients and controls are shown in Table 1. The age range of the subjects was 30–95 years. The median time of blood draw was 36±29 hours post stroke. Stroke was diagnosed according to World Health Organization stroke criteria.

**Table 1:** Clinical and laboratory characteristics of patients and controls.

Factor	All (n = 69)	Stroke (n = 43)	Control (n = 26)	p
Age	63.0 (52.0, 71.0)	70.0 (60.0, 73.0)	55.5 (50.2,61.0)	0.0002
Gender– male	28 (40)	18 (42)	10 (38)	0.9
Race– black	63 (91)	43 (93)	23 (88)	0.8
Risk factors				
Hypertension	54 (78)	41 (95)	13 (50)	<0.0001
Diabetes	24 (35)	16 (37)	8 (31)	0.8
Coronary artery disease	17 (25)	13 (30)	4 (15)	0.3
Smoking history	33 (48)	11 (25)	22 (85)	<0.0001
Atrial fibrillation	6 (9)	6 (14)	0 (0)	0.12
Hyperlipidemia	29 (42)	19 (44)	10 (38)	0.8
Medications				
Diuretics	13 (20)	11 (27)	2 (8)	0.10
ACEIs/ARBs	20 (30)	18 (45)	2 (8)	0.003
Beta blockers	29 (44)	22 (55)	7 (27)	0.046
Calcium channel blockers	18 (27)	14 (35)	4 (15)	0.14
Antithrombotics	28 (42)	20 (50)	8 (31)	0.2
Statins	24 (36)	16 (40)	8 (31)	0.6
Stroke-Related				
Infarct volume (mm^3^)	N/A	2,367.3 (1,028.4, 14,491.5)	N/A	N/A
NIHSS score	N/A	5.0 (3.0, 9.0)	N/A	N/A
Barthel score at 3–6 months	N/A	95.0 (87.5, 100)	N/A	N/A

Results are median (interquartile range) for continuous factors and numbers (%) for categorical factors. ACEI – angiotensin converting enzyme inhibitor, ARB - angiotensin receptor blocker, N/A – not applicable, NIHSS – National Institutes of Health Stroke Scale, Wilcoxon rank sum tests were used to compare the ages between the stroke patients and the control subjects. Chi–square and Fishers' exact tests were used to compare the remaining demographic, risk factors and medications between the stroke patients and the control subjects.

The study inclusion criteria were: over 18 years of age and acute ischemic stroke. The exclusion criteria were: current immunological diseases, taking steroid or immunosuppressive therapies, severe allergies, acute infection and severe anemia. The following clinical data were recorded: age, gender, race, self–reported risk factors, medications at the time of stroke onset (based on 40 subjects) or, in the control subjects, medications at the time of blood draw, National Institutes of Health Stroke Scale (NIHSS) score in the stroke subjects and complete blood counts (CBC), including total white blood cell count and white cell differential counts. Hypertension was defined as a prior (at any time in the past) diagnosis of hypertension by the subject's physician or currently receiving treatment for hypertension. Diabetes was defined as a past medical history of known diabetes mellitus. Coronary artery disease was defined as a physician-diagnosed past history of ischemic heart disease or angina. Hyperlipidemia was defined as a past history of documented elevation in total cholesterol (>200 mg/dl). Smoking was defined as current or prior smoking. Atrial fibrillation was defined as a past or current history of physician-diagnosed atrial fibrillation.

In the stroke patients lesion volumes were independently measured by two observers with neuroimaging expertise who were blinded to the clinical and laboratory data. Ischemic lesion volumes were measured on magnetic resonance imaging diffusion weighted images using Medical Image Processing Analysis and Visualization software. Neurological severity was based on the admission National Institutes of Health Stroke Scale (NIHSS) score. Follow–up assessment used the Barthel scores at 3 to 6 months (based on 33 patients) that provides a measure of the amount of stroke recovery and disability.

### Flow Cytometry

**Figure 1:**
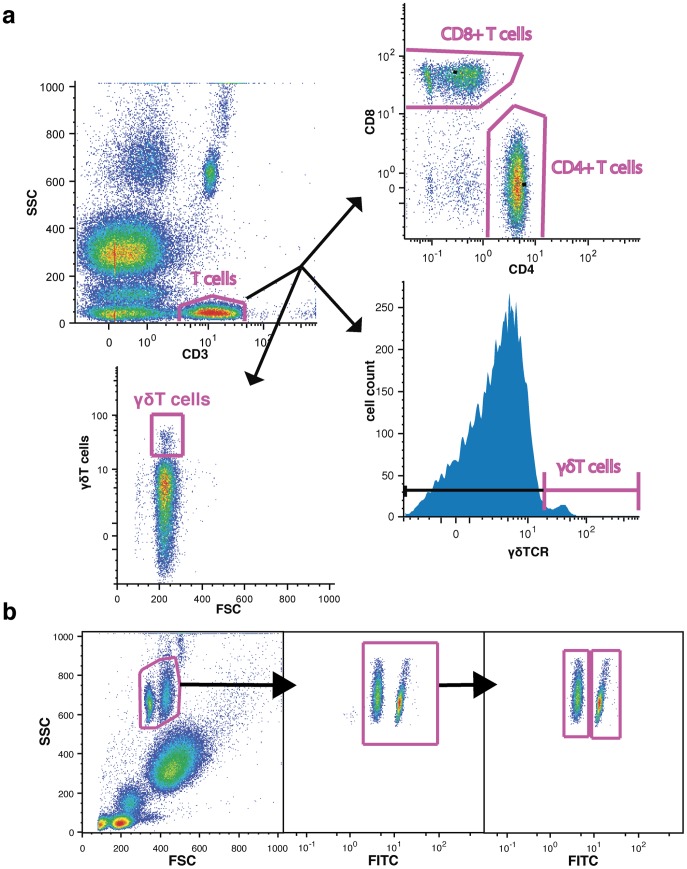
Gating strategy to identify T cell subsets (CD4+, CD8+ and γδTCR+) and counting beads based on a representative sample. (a) Gating strategy to identify T cell subsets. T lymphocytes were selected based on CD3 expression and side scatter (SSC) characteristics. The CD3+ population was further divided into CD4+/CD8+ T lymphocytes (based on CD4 and CD8 expression) and γδT positive and negative CD3 cells (based on γδTCR expression). (b) Gating strategy to identify counting beads. First, two populations of counting beads were selected based on the forward and side scatter characteristics. Second, FITC positive beads were counted for the cell count analyses. Third, the frequency of each bead population was assessed to compare with bead lot characteristics.

Peripheral blood from the study participants was drawn to BD Vacutainer tubes containing ethylenediaminetetraacetate (EDTA). Within an hour from blood collection 100 µl of blood was stained in the dark for 30 minutes in room temperature with four monoclonal antibodies (Ab) to CD3, CD4, CD8 and γδTCR (Table 2). Following staining, erythrocytes were lysed and leukocytes were fixed according to manufacturer No–Wash Lysis Procedure (Cal–Lyse solution Invitrogen). Counting beads (AccuCheck Counting Beads Invitrogen) were added, following manufacturer protocol, to obtain absolute cell counts (gating strategy presented in the Figure 1). Data were collected on the Epics XL flow cytometer (Beckman Coulter). Three populations of T lymphocytes were analyzed: CD8+, CD4+ and γδTCR+. T lymphocytes were defined as CD3+ cells within the lymphocyte gate. The lymphocyte gate was set based on side scatter characteristics. Gating strategy was set to include CD3+ cells regardless of size, not to exclude activated (larger) and apoptotic (smaller) lymphocytes (Figure 1). Based on forward scatter (FSC) values, size of CD3+ lymphocytes was not different from the size of γδT cells (mean ±standard deviation, respectively: 182±14 and 187±16; p>0.05) as presented on FCS vs. γδTCR+ plot (Figure 1). γδT cells size did not differ between stroke and control (mean ±standard deviation, respectively: 188±17 and 184±14; p>0.05). 200,000 events were acquired for each sample. Raw data were compensated, analyzed and presented using FlowJo software version 9.2 for Mac (Tree Star, San Carlos, California). Ab were titrated to achieve optimal resolution and gates for CD8+, CD4+ and γδTCR+ T lymphocytes were set using fluorescence minus one (FMO) method [19].

**Table 2:** Monoclonal antibodies.

Target	Antibody clone	Fluorophore	Volume used	Catalogue number	Manufacturer
CD3	S4.1	PE–Texas Red	0.5 µl	MHCD0317	Invitrogen
CD4	RPA-T4	FITC	2 µl	555346	BD Biosciences
γδTCR	11F2	PE	2 µl	340887	Becton Dickinson
CD8	HIT8a	PE–Cy5	2 µl	555636	BD Pharmingen

### Cytokine Levels and Gene Expression in γδT cells

γδT cells were extracted from 35 study subjects (17 stroke, 18 control). A detailed protocol for γδT cell sorting has been published previously [20]. Briefly, γδT cells were extracted from whole blood using magnetic bead separation (Miltenyi Biotec) – γδT cell purity was over 80%. RNA and proteins were extracted using column separation (Norgen). Quality and concentration of RNA and protein was measured using Qubit 1.0 Fluorometer (Invitrogen). Protein concentration was measured in 28 study subjects (10 stroke, 18 controls). The concentration of IL-17A and INF-γ in proteins extracted from γδT cell was determined by flow cytometry using BD cytometric bead array. Samples processing was performed according to the manufacturers instructions (BD Bioscience) using Human IL-17A Flex Set and Human IFN-γ Flex Set. Results were analyzed using FCAP Array version 3 software (BD Bioscience). Gene expression was measured in 29 study subjects (17 stroke, 12 control). RNA reverse transcription PCR was based on random hexamers (Life Technologies). The primers were designed using NCBI/Primer-Blast (Primer3 and BLAST), and wet tested. High throughput real time PCR run on BioMark HD system (Fluidigm) was used to measure gene expression of IL-23R and IL-23A genes. To compare gene expression between samples relative analytic approach was used. The relative analysis was based on the cell count input for each sample and was normalized to commercial cDNA (Universal cDNA Reverse Transcribed by Random Hexamer: Human Normal Tissues; Biochain, Newark, CA). Subjects selected for cytokine expression and gene expression were representative for the entire group used for cell phenotyping.

### Statistical Analyses

The data were analyzed using R version 2.15.2 [21]. Non–parametric and parametric tests, as appropriate, were used to compare demographic and laboratory values between the stroke and control subjects (Wilcoxon rank sum and Student's *t*, tests).Spearman correlation coefficients were used for correlational analyses. Chi–square and Fishers' exact tests were used to compare grouped data. The hierarchical cluster analysis used Ward's method and log transformed and normalized cell count data.

A factorial analysis of variance (section 4 of the Results) was used to evaluate for potential interactions in the associations of stroke and hypertension on γδT cell subset counts. Levene's tests confirmed homogeneity of the variances of the independent factors. A linear regression analysis was performed to adjust for baseline differences between patients and controls and to control for major potential confounders in the associations of hypertension and stroke with γδT cell counts. Measurements of the global validation statistic, skewness, kurtosis, link function and heteroscedasticity confirmed that all model assumptions were met. The γδT cell count data were log transformed for this analysis. P values <0.05 were considered statistically significant.

## Results

### 1. Reduction of γδT cell numbers in human ischemic stroke

The absolute number of circulating γδT cells was reduced in the stroke subjects relative to the controls, by almost 50% (p = 0.005, **Table 3**). In the stroke patients the median γδT cell count was 79.4 cells/µl while in the control subjects the median number of γδT cells was 137.0 cells/µl. γδT cells accounted for a median of 1.92% (range 0.28–22.9%) of CD3^+^ lymphocytes.

**Table 3:** Leukocyte subset cellular counts in stroke and control subjects.

	All (n = 69)	Stroke (n = 43)	Control (n = 26)	p
γδ^+^T (cells/µl)	98.6 (52.8, 197.0)	79.4 (45.7, 163.0)	137.0 (85.4, 254.5)	0.005
CD4^+^T (cells/µl)	3972.0 (2471.0, 5252.0	3903.0 (2641.0, 4835.0)	4346.5 (2430.5, 6862.0)	0.14
CD8^+^T (cells/µl)	1463.0 (950.0, 2094.0)	1343.0 (876.5, 1799.0)	1737.5 (993.7, 2487.7)	0.08
Neutrophils (10^9^/ml)	3.6 (2.8, 4.3)	3.7 (3.1, 4.8)	2.9 (2.4, 4.0)	0.045
Monocytes (10^9^/ml)	0.4 (0.3, 0.5)	0.4 (0.3, 0.5)	0.4 (0.3, 0.5)	0.99
Lymphocytes (10^9^/ml)	1.7 (1.3, 2.0)	1.6 (1.0, 1.9)	1.9 (1.5, 2.2)	0.026
Total WBC (10^9^/ml)	5.8 (4.8,7.3)	5.8 (5.2, 7.4)	5.5 (4.3, 6.8)	0.3

Values are median (interquartile range). γδ^+^T, CD4^+^T and CD8^+^T count was measured with flow cytometry; Neutrophil, Monocyte, Lymphocyte and Total WBC was measured with complete blood count; WBC – white blood cell. Wilcoxon rank sum tests were used to compare leukocyte cellular counts between the stroke patients and the control subjects.

**Figure 2:**
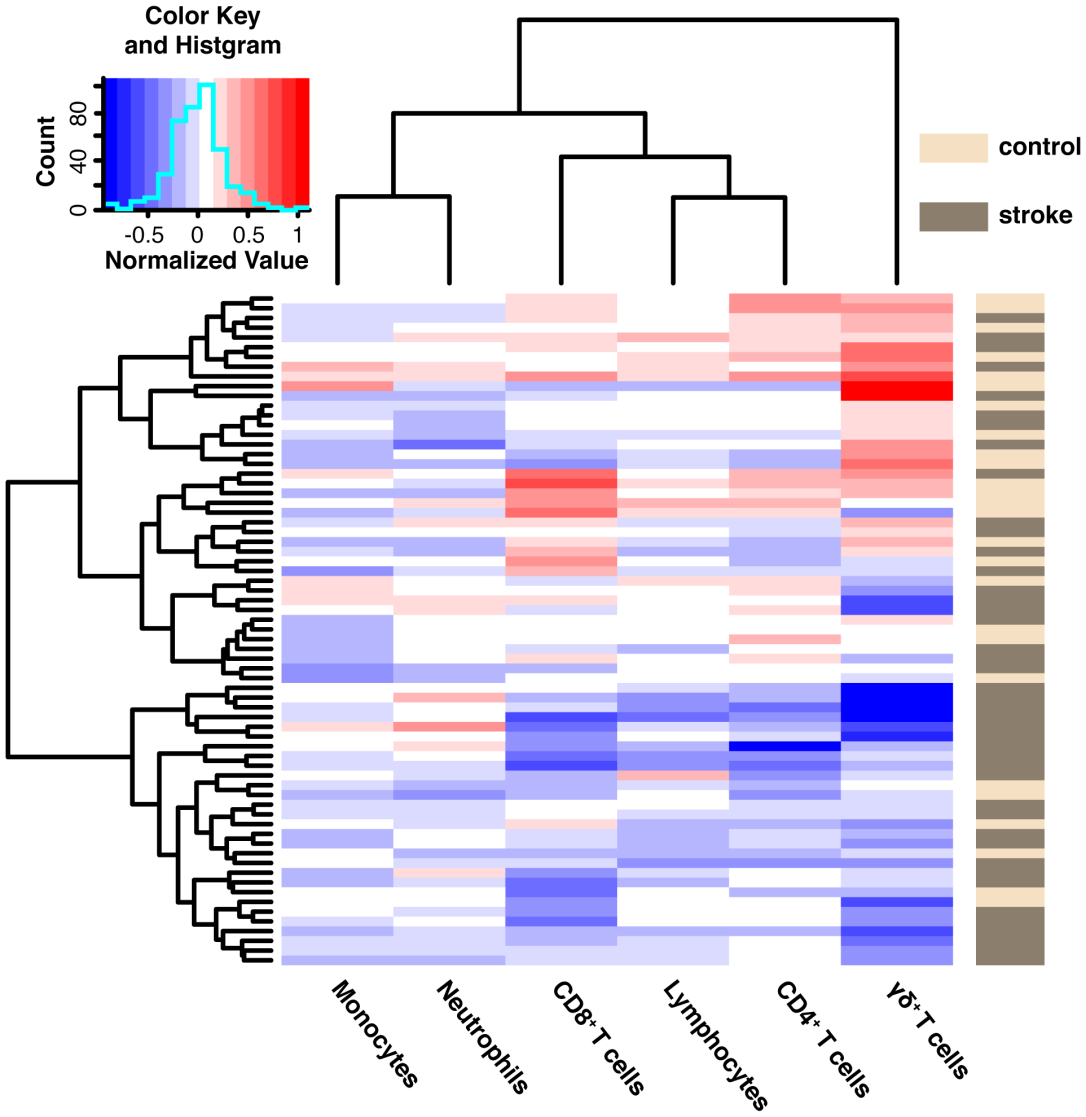
γδT cells were differentially altered in this clinical cohort relative to 5 other leukocyte subsets. This heatmap illustrates the normalized cell counts for 6 leukocyte cell subsets (columns) in the study subjects (rows). Cell numbers for neutrophils, monocytes and total lymphocytes were determined from the complete blood count, and cell numbers for CD4^+^, CD8^+^ and γδ^+^ T lymphocytes were measured using flow cytometry. The column dendrogram reveals clustering by leukocyte subset, demonstrating that the γδT cell subset formed a separate and dominant cluster from the other five leukocyte subsets. The γδT cell cluster separates into decreased and increased cellular counts, with decreased cellular counts predominating in the stroke subjects. This hierarchical cluster analysis used Ward's method and log-transformed and normalized cell count data.

γδT cells were disproportionately altered relative to the five other leukocyte subsets studied (**Figure 2**). Mild increases in neutrophils (p = 0.045) and decreases in the total number of lymphocytes (p = 0.03) were present in the ischemic stroke patients, while CD4^+^ and CD8^+^ T cells were non-significantly reduced (**Table 3**) and monocyte counts were unaltered (p = 0.99, **Table 3**).

### 2. γδT cell numbers did not correlate with lesion volume or clinical severity

There was no association of γδT cell count with ischemic lesion volume on DW MRI (coefficient  = −0.03, p = 0.9). There was also no association of γδT cell number with stroke severity as measured by the NIHSS (coefficient  = −012, p = 0.4) or with outcome (coefficient  = 0.02, p = 0.9).

### 3. Correlation of γδT cell numbers with demographic and risk factors

γδT cell numbers were decreased in individuals over age 60 years (p = 0.004) and in individuals with a prior history of coronary artery disease (p = 0.03). There was no association of γδT cell count with race or gender (**Table 4**). There was a substantial decrease in γδT cell counts in individuals with pre-existing hypertension. γδT cell counts were reduced by over 60% in hypertensive subjects (80.6 cells/µl) relative to normotensive subjects (217.0 cells/µl, p = 0.0005, **Table 4**). γδT cell counts were reduced in patients taking antithrombotics (p = 0.03) or β-blockers (p = 0.02) but not altered in patients taking angiotensin converting enzyme (ACE) inhibitors, angiotensin II receptor blockers (ARB), diuretics or calcium channel blockers.

**Table 4:** Associations of γδT cell counts with demographic and risk factors for all study subjects (n = 69).

γδT Cell Counts (cells/µl)			
	Factor Present	Factor Absent	p
Demographic Factors			
Age≥60 years	72.5 (29.8, 171.7)	130.0 (94.1, 238.0)	0.004
Gender - male	96.0 (59.0, 159.7)	102.0 (50.3, 199.0)	0.8
Race - black	102.0 (52.8, 193.5)	77.9 (53.4, 171.7)	0.8
Risk Factors			
Hypertension	80.6 (49.8, 148.5)	217.0 (126.5, 293.0)	0.0005
Diabetes	90.7 (55.6, 195.2)	98.6 (58.2, 197.0)	0.97
Coronary artery disease	59.9 (27.6, 141.0)	109.5 (64.8, 211.0)	0.03
Smoking history	120 (69.2, 209.0)	87.5 (42.2, 171.7)	0.08
Atrial fibrillation	90.2 (55.1, 172.4)	102.0 (52.8, 193.5)	0.7
Hyperlipidemia	102.0 (56.5, 171.0)	97.3 (50.1, 222.2)	0.9
			
Medications			
Diuretics	102.0 (69.2, 182.0)	96.1 (50.7, 199.0)	0.73
ACEIs/ARBs	87.5 (49.8, 169.2)	109.5 (55.8, 217.2)	0.34
Beta blockers	72.7 (29.3, 157.5)	130.0 (73.5, 228.0)	0.02
Calcium channel blockers	72.5 (51.5, 176.2)	105.0 (52.4, 211.2)	0.32
Antithrombotics	72.5 (46.0, 141.0)	125.0 (70.1, 233.0)	0.03
Statins	97.6 (65.0, 176.0)	102.0 (47.0, 215.0)	0.81

Values are median (interquartile range). Wilcoxon rank sum tests were used to compare γδT cellular counts with demographics, risk factors and medication usage.

To further explore this finding, the cellular counts of the other T cell subsets were compared between subjects with and without hypertension. The CD8^+^ subset (p = 0.01) was moderately reduced in individuals with hypertension while there was no significant difference in CD4^+^ subset numbers in the subjects with hypertension (**Table 5**). γδT cell numbers were compared between the hypertensive and normotensive controls where substantial reductions were found- 81.8 cells/µl in hypertensive control subjects compared to 238.0 cells/µl in the normotensive control subjects (p = 0.0006, **Table 5**). This was not explained by age differences, as the median age was not significantly different between the control subjects with and without hypertension (58.0 vs. 52.0 years, respectively). CD8^+^ T cells were non–significantly decreased in hypertensive control subjects relative to normotensive controls (**Table 5**).

**Table 5:** T cell numbers in subjects with and without pre-existing hypertension.

Subjects	γδT (cells/µl)	CD4 (cells/µl)	CD8 (cells/µl)
**All Subjects**			
Normotension (n = 15)	217.0 (126.5, 293.0)	4394.0 (2495.0, 7399.0)	2168.0 (1503.0, 3564.5)
Hypertension (n = 54)	80.6 (49.8, 148.5)	3885.5 (2458.7, 4865.7)	1238.5 (868.2, 1765.2)
p	0.0005	0.14	0.008
**Control Subjects**			
Normotension (n = 13)	238.0 (199.0, 316.0)	5427.0 (2471.0, 7682.0)	2168.0 (1657.0, 4064.0)
Hypertension (n = 13)	81.8 (59.9, 111.0)	3778.0 (2417.0, 5524.0)	1230.0 (950.0, 1754.0)
p	0.0006	0.5	0.1
**Stroke Subjects**			
Normotension (n = 2)	73.1 (61.7, 84.6)	3680.5 (3534.8, 3826.2)	2207.0 (1778.0, 2636.0)
Hypertension (n = 41)	79.4 (45.3, 171.0)	3903.0 (2588.0, 4856.0)	1247.0 (860.0, 1769.0)
p	0.8	0.9	0.27

Values are median (interquartile range). Wilcoxon rank sum test were used for the comparisons in T cell numbers between the subject groups.

### 4. Relative contributions of hypertension and stroke on γδT cell counts

In a factorial analysis of variance hypertension was the major determinant of γδT cellular counts (F value = 12.8; p = 0.0006). Stroke and hypertension:stroke interaction were not significant (respectively, F value = 1.54; p = 0.22 and F value = 2.1; p = 0.15). In linear regression analysis, adjusting for age, race and gender, pre–existing hypertension was also the most significant predictor of γδT cell counts (p = 0.008, **Table 6**). The estimated effect size of hypertension from the model was 2.77, indicating that hypertension is associated with a 64% reduction in γδT cell counts (**Table 6**). Coronary artery disease had no effect when entered into the model, nor did smoking history or beta-blocker or antithrombotic therapy.

**Table 6:** Linear regression model for γδT cell count.

Factor	Estimate	95% CI	T value	p
Age	−0.005	−0.04, 0.03	−0.02	0.80
Gender– male	0.12	−0.34, 0.58	0.53	0.60
Race– white	−0.04	−0.86, 0.77	−0.11	0.91
No hypertension	1.03	0.28, 1.78	2.73	0.008
Stroke	1.44	−1.08, 3.97	1.14	0.26
Hypertension:stroke interaction	−1.28	−2.87, 0.29	−1.62	0.11
Stroke:age interaction	−0.02	−0.06, 0.02	−1.13	0.26

Linear regression modeling was used to determine the relative impacts of hypertension and stroke on γδT cell counts and to adjust for clinical and demographic factors and potential interactions (for age, race and gender). This revealed that pre–existing hypertension was the significant predictor in the model accounting for γδT cell counts. Coronary artery disease had no effect when entered into the model, nor did smoking history, beta-blocker treatment, or antithrombotic treatment. CI– confidence intervals.

### 5. Cytokine levels and gene expression in γδT cells

In γδT cells there were increased levels of IL-17A in the stroke patients relative to the controls (p = 0.048) with a similar trend in IL-17A/IFN-γ ratio (p = 0.1) and with no alterations in IFN-γ levels (**Table 7**). There was a 3.3 fold non significant increase in *IL-23R* gene expression in γδT cells in the stroke patients (p>0.05) with a 1.1 fold non significant decrease in *IL-23A* gene expression (p>0.05). γδT cellular cytokine levels and gene expression were not significantly different between individuals with and without hypertension, with and without CAD and those over age 60 years relative to those less than age 60 years.

**Table 7:** γδT cellular cytokine levels (n = 28).

	Stroke	Control	p
IL-17A (ng/µl)*	7.01±2.3	4.78±3.1	0.048
IFN-γ (ng/µl)	2.4 (0.72, 3.58)	3.31 (1.43, 4.07)	0.4
IL-17A/IFN-γ ratio	3.3 (1.7, 6.2)	1.5 (0.9, 2.4)	0.1

Results are median (IQR) or mean* ± SD, as appropriate. Wilcoxon rank sum and Student's t tests, as appropriate, were used for the between group comparisons.

## Discussion

In this study we found that γδT cell numbers were reduced in human ischemic stroke and that γδT cells showed elevated IL-17A secretion. Reductions in γδT lymphocytes were also associated with hypertension, older age and, to a lesser degree, with prevalent coronary artery disease. Hypertension was associated with γδT cell count reductions of almost 65%. To the best of our knowledge this is the first clinical study showing the potential roles of γδT lymphocytes in stroke, hypertension and hypertension-mediated stroke.

In this study, the increase in IL-17A but not in INF-γ levels in the γδT cells of the stroke patients suggests activation of the IL-23 and IL-17 cytokine expression pathway. This is in line with previous reports by Shichita et al. from a murine stroke model and by Li et al. from human ischemic brain tissues [11], [22]. This increase in IL-17 expression from antigen-naive γδT cells may reflect a non-specific/antigen naive immune response to stroke [9], [10], [23]. Contrary to this murine study of Shichita et al. where γδT cell depletion ameliorated ischemia-reperfusion injury [11] we did not find associations of γδT cell counts with lesion volume, stroke severity and outcome. However, associations with lesion size and outcome and severity might be found in a larger patient sample.

Changes in leukocyte numbers, as reflected by elevated neutrophil counts and reductions in lymphocyte counts are common in acute stroke and reflect the phenomenon of adrenergic mediated stroke-induced immunodepression [24]. In stroke, activation of the sympathetic nervous system causes shrinkage of the spleen due to the release of residual cells [25]–[27] and is essential to the release of hepatic invariant NKT cells [6]. Blockade of the sympathetic nervous system has been shown to modulate circulating regulatory T cell numbers [28] and to change the activity of CD8^+^ and CD4^+^ T cells [29]. Stimulation of the sympathetic nervous system by acute stress has also been associated with increased numbers of circulating T cells expressing receptors for chemokines secreted by activated endothelial cells [30]. The reductions in γδT cells seen in this study may indicate that this is one of the populations of T lymphocytes that are regulated by the stress induced sympathetic response [6], [30]. Although, we did not specifically study the role of sympathetic activation in this study, we note that patients treated with β-blockers had significantly decreased numbers of circulating γδT cells.

The reductions of γδT cellular numbers in the stroke patients were disproportionately larger than reductions in the other lymphocyte subsets. Our results show that older age, hypertension and coronary artery disease were also associated with γδT cellular counts, with hypertension dominating γδT cell reductions. Older age has previously been reported to be associated with altered γδT cell counts [13], [14]. In CAD, no alterations in γδT cell counts have been reported to date, but decreases in CD3^+^ lymphocytes and increases of IFN–γ secreting CD8^+^CD56^+^ T lymphocytes and of CD28^—^CD4^+^ T lymphocytes - also a significant source of IFN–γ - have been reported [15]–[17]. Coronary artery infiltrating T lymphocytes producing IL–17 and IFN–γ [18] have been described. Given that γδT cells are a major source of both IL-17 and IFN–γ, our finding of decreased numbers of γδT cells in CAD is deserving of further study.

Hence, while the stroke patients and control subjects were matched on race and gender the subjects were not matched on age and hypertension status, which were associated with γδT cell counts. Given this finding, and given that most stroke patients are hypertensive, in order to fully and further explore the decrease in the gamma delta T cell counts in stroke patients stroke patients should be matched with controls based on their hypertension score.

Hypertension was overall the strongest determinant of γδT cell counts. Hypertension was the factor that was most strongly associated with γδT cell counts and was the most significant predictor in factorial and linear regression analyses. Markedly reduced cellular counts were also seen in the hypertensive control subjects, relative to the normotensive control subjects.

Hypertension is by far the most significant, modifiable risk factor for both ischemic and hemorrhagic stroke [31]–[33]. The high prevalence of hypertension in our non–Hispanic Black Caribbean stroke population is typical of many stroke cohorts. Hypertension has classically been considered a disorder of the renin angiotensin system or of the brain. Recently in rodent studies it has been demonstrated that T lymphocytes are required for the full development of angiotensin II induced hypertension [34]. T lymphocytes were found to infiltrate the adventitia and peri-adventitial fat of vessels [34] and T cells secreting IL–17 have been found in the vessel walls of hypertensive animals [34]–[36], potentially due to induction of hypertension-induced expression of homing receptors [35]. Furthermore, plasma and tissue levels of IL–17 and IFN–γ have been independently related with the development of hypertension, but the cellular sources were not identified [36]. As γδT cells secrete and are key sources of IL–17 and IFN–γ in the blood and IL–17 in the brain [4], [8], [37], the decrease in the number of circulating γδT cells in our study may be due to their active role in the evolution of hypertension and possible mobilization into vascular walls or their removal from the circulation. As the number of γδT cells distinguished the already diagnosed hypertensive subjects from non–hypertensive subjects, regardless of the medications being taken, as yet unrecognized pathophysiological mechanisms may be involved- other than targeted by current antihypertensive agents [38].

The implications of our results are that we have recognized two T lymphocyte subsets – the γδT and the CD8 subsets - that may be involved in human hypertension [39], with γδT cells possibly linking hypertension to stroke. γδT cells were identified for the first time as a potential marker of stroke risk in hypertensive individuals or of vascular disease burden. Alterations in CD8^+^ T lymphocytes support a recent finding where subsets of these cells were found to contribute to human hypertension [40]. The importance of these findings are highlighted by the fact that hypertension is the most common vascular disease worldwide with 5–30% of individuals remaining resistant to available treatments [31]. Hypertension–related morbidity comes largely from its downstream vascular consequences that include atherosclerotic vascular disease, heart disease and stroke, with stroke being the preeminent complication. Elucidating as yet unrecognized mechanisms of both hypertension and stroke could give new perspectives to the understanding of these diseases and possibly lead to novel treatments. Furthermore, this study demonstrates the importance of incorporating vascular risk factors in stroke translational research studies.

## References

[1] Go AS, Mozaffarian D, Roger VL, Benjamin EJ, Berry JD, et al (2013) Heart disease and stroke statistics—2013 update: a report from the American Heart Association. Circulation 127: e6–e245 10.1161/CIR.0b013e31828124ad 23239837 PMC5408511

[2] Iadecola C, Anrather J (2011) The immunology of stroke: from mechanisms to translation. Nat Med 17: 796–808 10.1038/nm.2399 21738161 PMC3137275

[3] Macrez R, Ali C, Toutirais O, Le Mauff B, Defer G, et al (2011) Stroke and the immune system: from pathophysiology to new therapeutic strategies. Lancet Neurol 10: 471–480 10.1016/S1474-4422(11)70066-7 21511199

[4] Gelderblom M, Weymar A, Bernreuther C, Velden J, Arunachalam P, et al (2012) Neutralization of the IL-17 axis diminishes neutrophil invasion and protects from ischemic stroke. Blood 120: 3793–3802 10.1182/blood-2012-02-412726 22976954

[5] Yilmaz G, Arumugam TV, Stokes KY, Granger DN (2006) Role of T lymphocytes and interferon-gamma in ischemic stroke. Circulation 113: 2105–2112 10.1161/CIRCULATIONAHA.105.593046 16636173

[6] Wong CHY, Jenne CN, Lee W-Y, Léger C, Kubes P (2011) Functional Innervation of Hepatic iNKT Cells Is Immunosuppressive Following Stroke. Science 334: 101–105 10.1126/science.1210301 21921158

[7] Liesz A, Suri-Payer E, Veltkamp C, Doerr H, Sommer C, et al (2009) Regulatory T cells are key cerebroprotective immunomodulators in acute experimental stroke. Nat Med 15: 192–199 10.1038/nm.1927 19169263

[8] Roark CL, Simonian PL, Fontenot AP, Born WK, O'Brien RL (2008) gammadelta T cells: an important source of IL-17. Curr Opin Immunol 20: 353–357 10.1016/j.coi.2008.03.006 18439808 PMC2601685

[9] Jensen KDC, Su X, Shin S, Li L, Youssef S, et al (2008) Thymic selection determines gammadelta T cell effector fate: antigen-naive cells make interleukin-17 and antigen-experienced cells make interferon gamma. Immunity 29: 90–100 10.1016/j.immuni.2008.04.022 18585064 PMC2601709

[10] Nakamura R, Shibata K, Yamada H, Shimoda K, Nakayama K, et al (2008) Tyk2-signaling plays an important role in host defense against Escherichia coli through IL-23-induced IL-17 production by gammadelta T cells. J Immunol 181: 2071–2075. 18641345 10.4049/jimmunol.181.3.2071

[11] Shichita T, Sugiyama Y, Ooboshi H, Sugimori H, Nakagawa R, et al (2009) Pivotal role of cerebral interleukin-17-producing gammadeltaT cells in the delayed phase of ischemic brain injury. Nat Med 15: 946–950 10.1038/nm.1999 19648929

[12] Peterfalvi A, Molnar T, Banati M, Pusch G, Miko E, et al (2009) Impaired function of innate T lymphocytes and NK cells in the acute phase of ischemic stroke. Cerebrovasc Dis 28: 490–498 10.1159/000236527 19752550

[13] Roux A, Mourin G, Larsen M, Fastenackels S, Urrutia A, et al (2013) Differential impact of age and cytomegalovirus infection on the γδ T cell compartment. J Immunol 191: 1300–1306 10.4049/jimmunol.1202940 23817410

[14] Andreu-Ballester JC, García-Ballesteros C, Benet-Campos C, Amigó V, Almela-Quilis A, et al (2012) Values for αβ and γδ T-lymphocytes and CD4+, CD8+, and CD56+ subsets in healthy adult subjects: assessment by age and gender. Cytometry B Clin Cytom 82: 238–244 10.1002/cyto.b.21020 22539222

[15] Brunetti ND, D'Antuono C, Rana M, D'Arienzo G, De Gennaro L, et al (2012) Lymphocyte subset characterization in patients with early clinical presentation of coronary heart disease. J Thromb Thrombolysis 34: 475–482 10.1007/s11239-012-0761-3 22903683

[16] Kolbus D, Ljungcrantz I, Andersson L, Hedblad B, Fredrikson GN, et al (2013) Association between CD8+ T-cell subsets and cardiovascular disease. J Intern Med 274: 41–51 10.1111/joim.12038 23356723

[17] Dumitriu IE, Baruah P, Finlayson CJ, Loftus IM, Antunes RF, et al (2012) High levels of costimulatory receptors OX40 and 4-1BB characterize CD4+CD28null T cells in patients with acute coronary syndrome. Circ Res 110: 857–869 10.1161/CIRCRESAHA.111.261933 22282196

[18] Eid RE, Rao DA, Zhou J, Lo SL, Ranjbaran H, et al (2009) Interleukin-17 and interferon-gamma are produced concomitantly by human coronary artery-infiltrating T cells and act synergistically on vascular smooth muscle cells. Circulation 119: 1424–1432 10.1161/CIRCULATIONAHA.108.827618 19255340 PMC2898514

[19] Herzenberg L a, Tung J, Moore W a, Herzenberg L a, Parks DR (2006) Interpreting flow cytometry data: a guide for the perplexed. Nat Immunol 7: 681–685 10.1038/ni0706-681 16785881

[20] Adamski MG, Li Y, Wagner E, Yu H, Seales-Bailey C, et al (2013) Next-Generation qPCR for the High-Throughput Measurement of Gene Expression in Multiple Leukocyte Subsets. J Biomol Screen 18: 1008–1017 10.1177/1087057113489882 23690294

[21] RDevelopment C (2012) TEAM (2008): R: A language and environment for statistical computing. Vienna, Austria. Internet http//www R-project org.

[22] Li G-Z, Zhong D, Yang L-M, Sun B, Zhong Z-H, et al (2005) Expression of interleukin-17 in ischemic brain tissue. Scand J Immunol 62: 481–486 10.1111/j.1365-3083.2005.01683.x 16305645

[23] Shibata K, Yamada H, Hara H, Kishihara K, Yoshikai Y (2007) Resident Vdelta1+ gammadelta T cells control early infiltration of neutrophils after Escherichia coli infection via IL-17 production. J Immunol 178: 4466–4472. 17372004 10.4049/jimmunol.178.7.4466

[24] Dirnagl U, Klehmet J, Braun JS, Harms H, Meisel C, et al (2007) Stroke-induced immunodepression: experimental evidence and clinical relevance. Stroke 38: 770–773 10.1161/01.STR.0000251441.89665.bc 17261736

[25] Sahota P, Vahidy F, Nguyen C, Bui T-T, Yang B, et al (2013) Changes in spleen size in patients with acute ischemic stroke: a pilot observational study. Int J Stroke 8: 60–67 10.1111/ijs.12022 23279654

[26] Offner H, Vandenbark AA, Hurn PD (2009) Effect of experimental stroke on peripheral immunity: CNS ischemia induces profound immunosuppression. Neuroscience 158: 1098–1111 10.1016/j.neuroscience.2008.05.033 18597949 PMC2666964

[27] Yu H, Adamski MG, Wagner E, Seales-Bailey C, Baird AE (2013) Splenic measurements in ischemic stroke: assessment of baseline size. Int J Stroke 8: E57 10.1111/ijs.12175 24267910

[28] Bhowmick S, Singh A, Flavell RA, Clark RB, O'Rourke J, et al (2009) The sympathetic nervous system modulates CD4(+)FoxP3(+) regulatory T cells via a TGF-beta-dependent mechanism. J Leukoc Biol 86: 1275–1283 10.1189/jlb.0209107 19741161 PMC2780915

[29] Grebe KM, Hickman HD, Irvine KR, Takeda K, Bennink JR, et al (2009) Sympathetic nervous system control of anti-influenza CD8+ T cell responses. Proc Natl Acad Sci U S A 106: 5300–5305 10.1073/pnas.0808851106 19286971 PMC2664017

[30] Bosch JA, Berntson GG, Cacioppo JT, Dhabhar FS, Marucha PT (2003) Acute stress evokes selective mobilization of T cells that differ in chemokine receptor expression: a potential pathway linking immunologic reactivity to cardiovascular disease. Brain Behav Immun 17: 251–259. 12831827 10.1016/s0889-1591(03)00054-0

[31] O'Donnell MJ, Xavier D, Liu L, Zhang H, Chin SL, et al (2010) Risk factors for ischaemic and intracerebral haemorrhagic stroke in 22 countries (the INTERSTROKE study): a case-control study. Lancet 376: 112–123 10.1016/S0140-6736(10)60834-3 20561675

[32] Kengne AP, Patel A, Barzi F, Jamrozik K, Lam TH, et al (2007) Systolic blood pressure, diabetes and the risk of cardiovascular diseases in the Asia-Pacific region. J Hypertens 25: 1205–1213 10.1097/HJH.0b013e3280dce59e 17563533

[33] Lewington S, Clarke R, Qizilbash N, Peto R, Collins R (2002) Age-specific relevance of usual blood pressure to vascular mortality: a meta-analysis of individual data for one million adults in 61 prospective studies. Lancet 360: 1903–1913. 12493255 10.1016/s0140-6736(02)11911-8

[34] Guzik TJ, Hoch NE, Brown KA, McCann LA, Rahman A, et al (2007) Role of the T cell in the genesis of angiotensin II induced hypertension and vascular dysfunction. J Exp Med 204: 2449–2460 10.1084/jem.20070657 17875676 PMC2118469

[35] Kim CH, Vaziri ND (2005) Hypertension promotes integrin expression and reactive oxygen species generation by circulating leukocytes. Kidney Int 67: 1462–1470 10.1111/j.1523-1755.2005.00223.x 15780098

[36] Von Vietinghoff S, Ley K (2010) Interleukin 17 in vascular inflammation. Cytokine Growth Factor Rev 21: 463–469 10.1016/j.cytogfr.2010.10.003 21075042 PMC3005323

[37] Ribot JC, DeBarros A, Pang DJ, Neves JF, Peperzak V, et al (2009) CD27 is a thymic determinant of the balance between interferon-gamma- and interleukin 17-producing gammadelta T cell subsets. Nat Immunol 10: 427–436 10.1038/ni.1717 19270712 PMC4167721

[38] Chon H, Gaillard CAJM, van der Meijden BB, Dijstelbloem HM, Kraaijenhagen RJ, et al (2004) Broadly altered gene expression in blood leukocytes in essential hypertension is absent during treatment. Hypertension 43: 947–951 10.1161/01.HYP.0000123071.35142.72 15007037

[39] Touyz RM (2012) New insights into mechanisms of hypertension. Curr Opin Nephrol Hypertens 21: 119–121 10.1097/MNH.0b013e328350a50f 22257800

[40] Youn J-C, Yu HT, Lim BJ, Koh MJ, Lee J, et al (2013) Immunosenescent CD8+ T cells and C-X-C chemokine receptor type 3 chemokines are increased in human hypertension. Hypertension 62: 126–133 10.1161/HYPERTENSIONAHA.113.00689 23716586

